# Genetic diversity and population structure of genes encoding vaccine candidate antigens of *Plasmodium vivax*

**DOI:** 10.1186/1475-2875-11-68

**Published:** 2012-03-14

**Authors:** Stella M Chenet, Lorena L Tapia, Ananias A Escalante, Salomon Durand, Carmen Lucas, David J Bacon

**Affiliations:** 1 Parasitology Program, Naval Medical Research Unit No. 6, Lima, Peru; 2 Arizona State University, School of Life Sciences, Tempe, AZ, USA; 3 Center for Evolutionary Medicine and Informatics, The Biodesign Institute, Arizona State University, Tempe, AZ, USA; 4 Naval Research Laboratory, Washington, DC, USA

**Keywords:** Malaria, *Plasmodium vivax*, Vaccine candidates, Haplotypes

## Abstract

**Background:**

A major concern in malaria vaccine development is genetic polymorphisms typically observed among *Plasmodium *isolates in different geographical areas across the world. Highly polymorphic regions have been observed in *Plasmodium falciparum *and *Plasmodium vivax *antigenic surface proteins such as Circumsporozoite protein (CSP), Duffy-binding protein (DBP), Merozoite surface protein-1 (MSP-1), Apical membrane antigen-1 (AMA-1) and Thrombospondin related anonymous protein (TRAP).

**Methods:**

Genetic variability was assessed in important polymorphic regions of various vaccine candidate antigens in *P. vivax *among 106 isolates from the Amazon Region of Loreto, Peru. In addition, genetic diversity determined in Peruvian isolates was compared to population studies from various geographical locations worldwide.

**Results:**

The structured diversity found in *P. vivax *populations did not show a geographic pattern and haplotypes from all gene candidates were distributed worldwide. In addition, evidence of balancing selection was found in polymorphic regions of the *trap, dbp *and *ama-1 *genes.

**Conclusions:**

It is important to have a good representation of the haplotypes circulating worldwide when implementing a vaccine, regardless of the geographic region of deployment since selective pressure plays an important role in structuring antigen diversity.

## Background

Malaria is one of the major global public health problems that affect most tropical regions of the world. Even though *Plasmodium falciparum *is the most virulent, it is estimated that *Plasmodium vivax *produces around 80 to 300 million clinical cases per year [1]. Furthermore, there have been several reports of severe *P. vivax *malaria cases in the last few years [2-4]. In 2008, 560,221 malaria cases were reported in the Americas [5]; 74.2% of them caused by *P. vivax *and 25.7% by *P. falciparum *[1]. About 90% of these malaria cases originated in the Amazon basin shared by Bolivia, Brazil, Colombia, Ecuador, French Guiana, Guyana, Venezuela, Suriname and Peru [5], whereas the other 10% was contributed by non-Amazon regions.

Developing a vaccine for *P. vivax *represents a major challenge especially considering the lack of *in vitro *cultures. Thus, current efforts focus on orthologs of *P. falciparum*. Over the past four decades, experiments performed in animals and human subjects have led to the development of several *Plasmodium *vaccine candidates. Antigenic surface proteins such as the Circumsporozoite protein (CSP), Thrombospondin related anonymous protein (TRAP), Duffy-binding protein (DBP), Merozoite surface protein-1 (MSP-1) and Apical membrane antigen-1 (AMA-1) are currently being evaluated as vaccine candidates in clinical trials [1,6-8]. The CSP is one of the most extensively studied antigens and it is involved in the motility and invasion of the sporozoite during its entrance in the hepatocyte [9]. *Pv*CSP has an immunogenic central repeat domain flanked by amino and carboxyl sequences containing highly conserved protein stretches (Regions I and II-plus) [10]. This protein displays two major types of nonapeptide repeats: type I (VK210), which contains repeats of GDRA(A/D)GQPA or related sequences and type II (VK247), which is composed of ANGAGNQPG repeats [11,12]. A third type of variant, called *P. vivax*-like has repeat units of APGANQ(E/G)GAA, identical to that described for *Plasmodium simiovale *[13,14]. Currently, the *P. falciparum *RTS, S vaccine, which is based on CSP have showed to protect semi-immune adults [15] and children from *P. falciparum *natural infection in endemic areas [16-18]; this vaccine is being tested in Phase III clinical trials [8,19].

Another sporozoite surface protein that stands out as a good vaccine candidate is TRAP, which is a 90 kDa protein with six distinct regions. TRAP is essential for sporozoite gliding and hepatocyte invasion [20,21]. Genetic analysis of *Pv*TRAP reveals that most of the polymorphisms occur in Region II (von Willebrand factor A-domain) and III [22]. The potential role of TRAP as a malaria vaccine candidate has been tested in mice and monkeys, inducing high levels of specific antibodies [23]. Furthermore, a synthetic peptide-derived from a conserved region of the TRAP protein located in the N-terminal, proved to be immunogenic and provided partial protection in *Aotus *monkeys [1,24].

On the other hand, the asexual *P. vivax *parasite antigens DBP, MSP-1 and AMA-1 have also received attention for their role in the invasion process and/or their generation of antibody response. DBP interacts with the Duffy blood group antigen of reticulocytes, MSP-1 is expressed abundantly on the merozoite surface and AMA-1 plays a role in apical reorientation of merozoites following initial attachment during invasion [1]. Region II of DBP (DBP_II_) functions as a ligand domain and consists of 330 aa with 12 cysteines and numerous aromatic residues. Studies have identified the central region of the DBP_II _domain between cysteines 4 and 8, as the segment containing the conserved contact residues Tyr 94, Asn 95, Lys 96, Arg 103 and Ile 175 required for recognition of DARC (Duffy Antigen/Receptor for Chemokine) on human erythrocytes [25-28].

MSP-1 is part of a complex of proteins that is processed into smaller fragments by serine proteases during invasion of red blood cells [29]. It has been suggested that fragments *Pv*MSP-1_14 _and *Pv*MSP-1_20 _could be included in a *P. vivax *multi-antigen vaccine since they contain high affinity reticulocyte binding (HARB) cluster regions and confer protection in monkeys [30-32]. AMA-1 has also been evaluated for inclusion in a multi- sub-unit vaccine for both *P. falciparum *and *P. vivax *[33,34]. The complete AMA-1 protein contains three domains, from which a higher rate of mutations and level of diversifying selection has been shown in domain I [35,36]. Indeed, AMA-1 is highly immunogenic, protecting against *Plasmodium chabaudi *infection in mice [37] and displaying significant antibody and T cell responses in endemic human populations [38-40].

Investigation of the sequence variation among malaria antigenic regions is necessary for the development of an effective malaria vaccine that includes haplotypes from different regions, considering that *Plasmodium *is highly diverse and these regions are under strong immunological pressure. In this study, important regions in *Pv*CSP, *Pv*TRAP, *Pv*DBP, *Pv*MSP-1 and *Pv*AMA-1 were genotyped using *P. vivax *Peruvian isolates collected during years 2006-2007. In addition, since natural selection plays a role in structuring diversity of these highly polymorphic antigens, the worldwide distribution of the main variants was determined by comparing the Peruvian samples with sequences previously reported from South America, Asia and Oceania.

## Methods

### Study area and sample collection

Blood samples from patients were collected during years 2006-2007 from endemic areas in the Peruvian Amazon region of Loreto Department using human use approved protocols (NMRCD.2005.0005). In Peru, *P. vivax *corresponds to more than 80% of the malaria cases and the majority of them are found in the Amazonic Department of Loreto [41]. Fifty-seven samples were obtained from Padrecocha, in Punchana district, a village situated five km from Iquitos (Loreto's capital) and forty-nine samples were collected from a village located in the San Juan district just south of Iquitos. All samples were confirmed to be positive for *P. vivax *by microscopic analysis on Giemsa-stained blood smears.

### PCR and DNA sequencing

DNA was purified from 200 μL of whole blood using the QIAamp DNA Blood mini kit (QIAGEN, Valencia, CA). PCR amplification was performed in a 50 μL reaction mixture containing 0.2 μM each of forward and reverse primers, 200 μM each dNTP, 0.6 units of DNA polymerase recombinant (Invitrogen, Carlsbad, CA), 3 μL of 10× PCR buffer, 1.5 mM of MgCl_2 _and 5 μL of DNA extracted from blood. To assess antigenic variability of *Pv*CSP, the Central Repetitive Region, Region I and Region II were amplified. The PCR conditions were: 5 min at 94°C; 1 min at 94°C, 1 min at 58°C, 2 min at 72°C for 35 cycles; and a final extension of 72°C for 2 min, using the primers VSC-OF (5'ATGTAGATCTGTCCAAGGCCATAAA3') and VSC-OR (5' TAATTGAATAATGCTAGGACTAACAATATG 3') [42]. The amplification of the highly polymorphic regions II and III of *Pv*TRAP [22] was obtained using the primers Pvsspf: 5' GTCGATTTATACCTCCTAGTTGACGG 3' and Pvsspr: 5' CAGCTTGAACGTCGACCTCCGC 3' with the following conditions: 5 min at 94°C; 1 min at 94°C, 1 min at 50°C, 2 min at 72°C for 35 cycles; and a final extension of 72°C for 2 min. The amplification of the central region of the *Pv*DBP_II _domain was done using primers Liz-1 (5' TGGGGAGGAAAAAGATG 3') and Liz-2 (5' AACGGAACAAACGCATAAC 3') [43]. The PCR conditions used were as follows: 4 min at 94°C; 1 min at 94°C, 2 min at 58°C, 2 min at 72°C for 35 cycles; and a final extension of 72°C for 3 min. *Pv*MSP-1 primers described by Espinosa et al. (HARB1_f: 5' GGCAACAACGATGACGAC 3', HARB1_r: 5' CTTCTCAAGTCGAGCAG 3', HARB2_f: 5' TCACTGTAAAGAAATTGCAG 3' and HARB2_r: 5' ATACATGTGTGCTCGGAG 3') were used for amplifying the two HARB containing regions: *Pv*MSP-1_20 _and *Pv*MSP-1_14 _kDa [31] with the following conditions: 5 min at 94°C; 1 min at 94°C, 1 min at 50°C, 1 min at 72°C for 35 cycles; and a final extension of 72°C for 5 min for the first region and 5 min at 94°C; 1 min at 94°C, 1 min at 45°C, 1 min at 72°C for 35 cycles; and a final extension of 72°C for 5 min for the second region. Finally, a nested PCR was used to amplify domain I of *Pv*AMA-1 as described by Cheng and Saul [44]. PCR products were purified using the QIAquick PCR purifications kit (QIAGEN, Valencia, CA, USA) and sequenced using BigDye^® ^Terminator v3.1 sequencing kit (Applied Biosystems, Foster City, CA, USA), with the primers previously mentioned and an ABI 3100 automated sequencer.

### Sequence determination and data analysis

Sequences were analysed using the Sequencher™ software version 4.7 (Gene Codes Corporation, Ann Harbor, MI, USA) and sequence alignments were performed using the MEGA software version 4.1 [45]. Comparison with previously deposited *P. vivax *sequences in the GenBank was performed using BLAST search. The number of haplotypes (h), haplotype diversity (hd) and nucleotide diversity (Pi) values were obtained using the DnaSP version 5.0 [46]. The effect of natural selection was also evaluated by estimating the difference of non-synonymous and synonymous substitutions (Dn-Ds) using the Nei and Gojobori's method with the Jukes and Cantor correction. The null hypothesis of neutrality was rejected if Dn:Ds ≠ 1. Positive values for Dn-Ds indicated positive selection while negative values indicated negative selection [47]. Standard error was calculated using bootstrap with 1,000 replications and rates of Ds and Dn were compared by the Z-test of selection using Mega version 4.1 [45]. To test whether the haplotypes clustered according to their geographic origin, the model-based clustering algorithm implemented in the Structure 2.1 software was used [48]. This software uses a Bayesian clustering approach to assign isolates to *K *populations characterized by a set of allele frequencies at each locus. The number of such populations may be either previously known or not. The program was run five times at different *K *values (from 2 to 10) with a burn-in period of 10,000 iterations followed by 50,000 iterations. The admixture model was used in all analyses, which allows for the presence of individuals with ancestry in two or more of the *K *populations. However, clustering algorithms incorporate stochastic simulation as part of the inference and, as a consequence, independent analyses of the same data may result in several distinct outcomes, even though the same initial conditions were used. To facilitate the interpretation of population-genetic clustering results, the computer program CLUMPP (Cluster Matching and Permutation Program) was used. CLUMPP strips away the 'label switching' heterogeneity so that the 'genuine multimodality' can be detected and quantified [49]. In addition, *distruct *1.1 was used to graphically display the clustering results [50]. The genetic difference between clusters was measured by the fixation index (Fst) calculated from the haplotype frequencies (Hst) and pair-wise DNA sequence diversity (Kst*) using DnaSP version 5.0 [46]. The nearest-neighbour statistic (Snn) was also calculated between geographic populations. Snn is a measure of how often the "nearest neighbours" of sequences are from the same locality in geographic space. This statistic is applicable when genetic data are collected on individuals sampled from two or more localities. Snn is expected to be near one when the populations at the two localities are highly differentiated and near one-half when the populations at the two localities are part of the same pan-mictic population [51].

## Results

### PvCSP

A 1,100 bp PCR product corresponding to *Pvcsp *was amplified from 106 *P. vivax *samples from Peru. In addition, 326 complete CSP sequences from Brazil, Colombia, India, Iran and Korea were also included in the amino acid alignment. Sequences from Thailand, China and PNG were used to determine the percentage of strains circulating of each *Pv*CSP type (Table 1); however, they were not considered in the haplotype analysis since the sequences were not complete. Most of the strains circulating worldwide belonged to the VK210 type and in most population sets analysed (except Colombia and Thailand) a greater percentage of VK210 haplotypes was found. The amino acid alignment of the sequences used in this study showed 97 different VK210 haplotypes and 28 VK247 haplotypes circulating worldwide. Different haplotypes were observed in South American and Asian countries, except for one VK247 Peruvian haplotype, which was also found in Iran [52]. Thirty-seven VK210 and 19 VK247 haplotypes were identified in South America; while in Asia, 51 and 10 haplotypes were observed from each type.

**Table 1:** Population data set for *Pv*CSP (* Submission year, ? partial sequences)

Region	Country	Year of collection	GenBank Accession Number	N	% VK210-VK247	H
South America	Brazil	2000	DQ978648-DQ978690	43	100 -0	16
	Colombia	2009	GU339059-GU339086	28	11.2-88.8	20

South Asia	India	2008*	FJ491063-FJ491141	79	100 -0	44

South East Asia	Thailand	2009*	HQ011279-HQ011323	45	40-60	?

East Asia	Korea	2006*	DQ859734-DQ859772	39	100-0	7
	China	2008*	FJ601724-FJ601761	38	100-0	?

Western Asia	Iran	2000-2003	AY367278-AY367303, AY632256-AY632287, AY443700-AY4443725, AY632288-AY632318, AY632243-AY632251, AY632319-AY632330, AY443726	137	76.5-23.5	25

Oceania	PNG	1999, 2001, 2003	EU031819-EU031836	17	70.6-29.4	?

**Figure 1:**
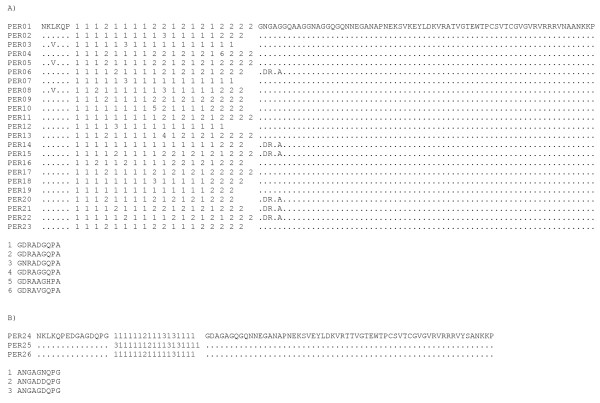
**Amino acid alignment of the 27 *Pv*CSP haplotypes bearing a) the VK210 repeat type and b) the VK247 repeat type in Peruvian samples**.

In Peru, 23 different VK210 subtypes and three different VK247 subtypes were found (Figure 1). *Pv*CSP displayed a different pattern of a nine amino acid oligopeptide repetitions depending on the subtype. The VK210 subtypes contained the repeat region G(D/N)RA(A/D/G/V)G(Q/H)PA either 16, 17, 18 or 19 times, while VK247 subtypes contained the repeated region ANGA(G/D)(D/N)QPG, 17 or 18 times. Region I of *Pv*CSP showed a polymorphism in position 3 (L/V), whereas the 18 amino acid long linear peptide EWTPCSVTCGVGVRVRRR, located on Region II [53,54], was highly conserved. *Pv*CSP analysis of Peruvian isolates also revealed high variability of VK210 and VK247 haplotypes compared to other populations from South America [55,56]. Region II-plus, which contains dominant B-cell epitopes [57,1] and block hepatocyte invasion by sporozoites [58], was highly conserved in Peruvian isolates, with the same amino acid sequence as the Sal I strain.

### PvTRAP

A total of 104 sequences of regions II and III of *Pvtrap *were obtained from the Peruvian isolates. In addition, 63 Thailand sequences from GenBank were included in the amino acid alignment. The N-terminal region (209-256aa), which has been proved to be immunogenic and to provide partial protection in *Aotus *monkeys [24] was highly conserved with only three amino acid substitutions in positions 216 (R/K), 219 (H/Q) and 229 (G/K). In general, more diversity was found in Thailand (h = 34, π = 0.007 ± 0.00029 and Hd = 0.961 ± 0.01) (Table 2) compared to Peru (h = 7, π = 0.00235 ± 0.00012 and Hd = 0.641 ± 0.026) and according to the Dn-Ds test, regions II and III of *Pvtrap *were under positive selection (Table 3).

**Table 2:** Estimates of *Plasmodium vivax *genetic diversity using GenBank sequences

Antigen	Region	Country	N	GenBank Accession Number	H	π (s.d.)	Hd (s.d.)
*TRAP*	South East Asia	Thailand	63	AF312559-AF312661	34	0.00707 (0.00029)	0.961 (0.01)

*DBPII*	South America	Brazil	126	DQ156520, EU812839-EU812960, EU870443-EU870445	24	0.00919 (0.00042)	0.883 (0.018)
	
		Colombia	17	DQ156513, U50575-U50590	12	0.00812 (0.00081)	0.956 (0.033)
	
	South Asia	India	101	DQ156516, FJ491142-FJ491241	28	0.01011 (0.00063)	0.86 (0.028)
	
		Sri Lanka	100	GU143914-GU144013	14	0.00864 (0.00063)	0.738 (0.039)
	
	South East Asia	Thailand	30	EF219451, EF368159-EF368178, EF379127-EF379135	19	0.01628 (0.00354)	0.972 (0.018)
	
	East Asia	Korea	16	EU395592, DQ156515, DQ156522-DQ156523, AF215737-AF215738, AF220657, AF220659-AF220667	11	0.00817 (0.00149)	0.933 (0.048)
	
	Western Asia	Iran	11	EU860428-EU860438	8	0.01047 (0.00205)	0.945 (0.054)
	
	Oceania	Papua New Guinea	200	AF291096, AF289653, AF469515-AF469602, DQ156512-DQ156523, AY970837-AY970925	59	0.01166 (0.00041)	0.888 (0.015)

*MSP-1*	South America	Brazil	8	AF435622-AF435625, AF435627, AF435629-AF435631	4	0.00293 (0.00061)	0.75 (0.139)
	
	South East Asia	India	28	EU430452-EU430479	6	0.0172 (0.00049)	0.489 (0.112)
	
		Thailand	170	GQ890872-GQ891041	2	0.00013 (0.00005)	0.068 (0.026)
	
		Singapore	50	GU971656-GU971705	10	0.0019 (0.00032)	0.407 (0.09)
	
	Western Asia	Turkey	30	AB564559-AB564588	2	0.00073 (0.00034)	0.186 (0.088)
	
	East Asia	Korea	8	HQ171941-HQ171934	1	0	0

*AMA-1*	South America	Brazil	65	EF031154-EF031216, EF031183, EF031184	12	0.01358 (0.00056)	0.866 (0.02)
	
		Venezuela	73	EU346015-EU346087	12	0.01368 (0.00053)	0.847 (0.019)
	
	South Asia	India	189	EU282774-EU282822, FJ490963-FJ491062, EF025187-EF025197, GU086369-GU086382, AF171250-AF171254, AF171256, AF171258-AF171260, AF171262-AF171267	68	0.01427 (0.00036)	0.945 (0.008)
	
		Sri Lanka	23	EF218679-EF218701	8	0.00722 (0.00148)	0.83 (0.054)
	
	East Asia	China	7	AF171243-AF171249	6	0.01211 (0.00193)	0.893 (0.111)
	
		Korea	6	AF357212, AF357213, GU476488, EU395599, AF402593, AF402594	4	0.00812 (0.00328)	0.9 (0.161)
	
	South East Asia	Myanmar	39	FJ157247-FJ157285	36	0.01833 (0.00092)	0.996 (0.007)
	
		Philippines	110	AF171273-AF171382	20	0.01474 (0.0005)	0.802 (0.029)
	
		Thailand	235	FJ784891-FJ85121, AF17453-AF171456	44	0.01488 (0.00039)	0.899 (0.011)
	
	Oceania	PNG	22	AF171421-AF171426, AF171428-AF171443	13	0.01379 (0.00163)	0.913 (0.045)
	
		Solomon Islands	5	AF171444, AF171445, AF171447, AF171449, AF171450	5	0.01855 (0.00318)	1 (0.126)

**Table 3:** Summary of genetic data of the *Plasmodium vivax *vaccine antigens analysed including Sal I sequence.

Antigen	Expression	Domain	Sites	N	H	π (s.d.)	Hd (s.d.)	dN-dS (s.d.)	Ztest (dN ≠ dS)
*Dbp*	Merozoite	Region II	510	707	150	0.01103 (0.00025)	0.9319 (0.0053)	0.009 (0.003)	*P *< 0.05
*Msp-1*	Merozoite	MSP1_14 _and MSP1_20_	512	400*	28	0.00129 (0.00014)	0.358 (0.031)	0.001 (0.001)	n.s.
*Ama-1*	Merozoite	Domain I	345	872	168	0.01653 (0.00016)	0.9691 (0.0017)	0.012 (0.004)	*P *< 0.05
*Trap*	Sporozoite	Regions	837	168	38	0.0059 (0.00029)	0.854 (0.018)	0.007 (0.002)	*P *< 0.05

*Belem sequence included

### PvDBP

A 1,150 bp DNA fragment of *Pvdbp *was amplified from all 106 Peruvian isolates and 601 *P. vivax *sequences from Brazil, Colombia, India, Sri Lanka, Thailand, Korea, Iran and Papua New Guinea were also included in the analysis. The alignment of 510 bp from region II of *dbp *showed 150 different haplotypes, from which 39 were found in South America, 42 in Asia, 56 in Oceania and 13 were found in South America and Asia. In the Peruvian isolates 26 different haplotypes were found with genetic diversity values of π = 0.008 ± 0.00048 and Hd = 0.903 ± 0.014. In general, the average genetic and haplotype diversity in South America and Asia was very similar (Table 2). On the other hand, the analysis of DNA sequence polymorphisms showed a significant difference between synonymous and non-synonymous substitutions, indicating balancing selection in *dbp_II _*(Table 3). In addition, several substitutions in the positions underlined were found in DBPII regions corresponding to peptides H1: FHRDITFRKLYLKRKL, H3: DEKAQQRRKQWWNESK an 1653: RDYVSELPTEVQKLKEKC, previously reported as immunogenic [27,59].

### PvMSP-1

According to the amino acid alignment of *PvMSP-1*_20 _and *PvMSP-1*_14_, a total of 28 haplotypes were found worldwide, 11 in South America, 14 in Asia and three in both continents. When comparing genetic diversity between populations, Peru (π = 0.00286 ± 0.00029, Hd = 0.665 ± 0.041) and Brazil (π = 0.00293 ± 0.00061, Hd = 0.75 ± 0.139) showed the highest levels of nucleotide and haplotype diversity, while in Korea, Thailand and Turkey these HARBs were highly conserved. In general, values of genetic diversity for *Pvmsp-1 *were low when compared with the other genes and no clear signature of selection was found according to the Dn-Ds test (Table 3).

### PvAMA-1

The polymorphic region of *Pvama-1 *(Domain I of protein) was amplified and sequenced from 98 of the 106 Peruvian isolates. A total of 774 *Pvama-1 *partial sequences were used in the analysis, including samples from Brazil, Venezuela, India, Sri Lanka, China, Korea, Myanmar, Philippines, Thailand, Papua New Guinea and Solomon Islands. A total of 168 haplotypes were identified; 135 were exclusively found in Asia, nine in Oceania and 12 in South America. In addition, seven haplotypes were shared in America and Asia and five in Asia and Oceania. According to the analysis of polymorphisms, the dN-dS test indicated balancing selection in this domain (Table 3) with several haplotypes in low frequency. In addition, values of nucleotide diversity were very similar between geographic regions and populations (Table 2).

### Population genetic analysis

When analysing *Plasmodium *isolates, samples are usually grouped by geographic location; nevertheless, this approach assumes that geographic barriers or distance are the underlying factors that limit gene flow. It also assumes that genetic exchange within each population geographically defined is random. To better estimate the distribution of haplotypes of different *P. vivax *vaccine candidate antigens, a Bayesian clustering algorithm [48] was used to sub-group samples according to related SNPs haplotypes. The Structure model assumes that loci are independent within populations; however, this assumption is likely to be violated for sequence data, or data from non-recombining regions. It is a matter of debate whether it is valid to group sequences containing potentially linked polymorphisms in a single gene under balancing selection [60]. The main cost of the dependence within regions will be that Structure underestimates the uncertainty in the assignment of particular individuals. However, if there is enough independence across regions than LD within regions, then Structure may actually perform reasonably well [48]. Moreover, our main goal is not to describe the demographic history of the population but to get a graphically representation of the vaccine candidate's haplotype distribution. Genetic differentiation between clusters was calculated using Fst from haplotype-frequencies and pairwise DNA sequence diversity. The haplotype frequency-based statistics are more sensitive for small sample sizes, while the sequence-based statistic is a more sensitive method for detecting population structure in highly polymorphic loci [61,62].

**Figure 2:**
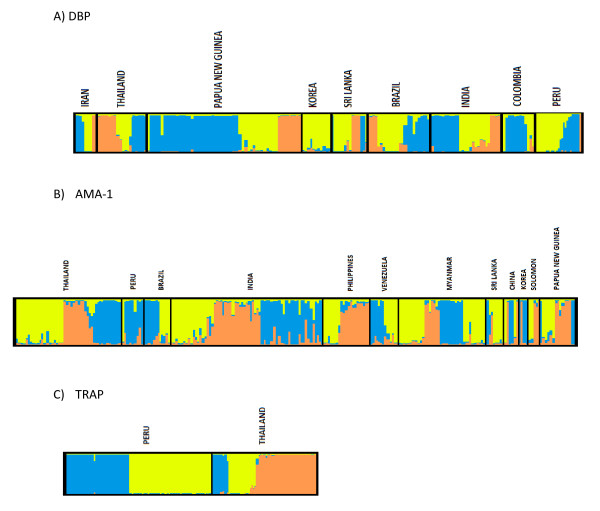
**Global population structure of the genes encoding *P.vivax *vaccine antigens based on Bayesian cluster analysis**. Each haplotype is represented by an imaginary bar, which is partitioned into K coloured segments that represent the membership fraction of each haplotype in K clusters (K = 3, represented by the three different colours blue, yellow and orange). Samples are grouped in boxes according to their geographic origin.

The results obtained with *Pv*TRAP, *Pv*DBP and *Pv*AMA-1 showed three different clusters. Significant differentiation was observed in haplotype and pair-wise comparisons between all clusters according to the Fst values (Table 4), except for *Pvmsp-1*, from which a significant number of clusters it was not possible to identify. Although admixed haplotypes were observed in *Pvdbp *and *Pvama-1*, the estimate of K = 3 best explained the distribution of haplotypes from all geographic populations; besides, the defined sub-groups were not geographically or even regionally restricted (Figure 2). Snn values between the different geographic populations were also calculated for each of the antigens. We observed that for most of the comparisons in DBP, AMA-1 (Table 5) and TRAP (Snn = 0.86803*), Snn values were near one, which indicates that the localities are highly differentiated. However, close geographic populations such as Sri Lanka and India in South Asia and Peru and Brazil in South America showed almost not genetic differentiation (Snn = 0.58, *p *< 0.01 and Snn = 0.65, *p *< 0.01). Thus, relative low Snn values were found between Brazil, India and Sri Lanka, since there are shared haplotypes circulating in South America and South Asia. Moreover, previous work also indicated low genetic differentiation using Fst analysis between *dbpII *sequences from Brazil and Iran, India or Sri Lanka [63]. On the other hand, at least seven of the pairwise comparisons made using MSP-1 resulted in non-significant values.

**Table 4:** Estimation of genetic differentiation between clusters of populations obtained by Structure.

A)			
**Antigen**	**1 vs 2**	**1 vs 3**	**2 vs 3**

**DBP**	0.13742*	0.18583*	0.11845*

**AMA-1**	0.09891*	0.06873*	0.06239*

**TRAP**	0.2478*	0.3393*	0.12982*

B)			

**Antigen**	**1 vs 2**	**1 vs 3**	**2 vs 3**

**DBP**	0.34601*	0.4555*	0.41168*

**AMA-1**	0.50583*	0.31637*	0.177725*

**TRAP**	0.71399*	0.60509*	0.64183*

*F*_ST _calculated from A) haplotype frequency and B) sequence diversity with *P < 0.001

**Table 5:** Estimation of genetic differentiation between geographic populations using the Snn statistics with *P < 0.01

A)										
	**Brazil**	**Colombia**	**Peru**	**India**	**Sri Lanka**	**Thailand**	**Korea**	**Iran**		
**Colombia**	0.93864*									
**Peru**	0.65322*	0.9486*								
**India**	0.63332*	0.94986*	0.74331*							
**Sri Lanka**	0.61225*	0.94952*	0.67073*	0.58268*						
**Thailand**	0.8613*	0.81977*	0.91711*	0.80463*	0.8734*					
**Korea**	0.92369*	0.91515*	0.90485*	0.9886*	0.80846*	0.80833*				
**Iran**	0.85693 ns	0.82143*	0.84720*	0.84885*	0.84003*	0.62883*	0.76684*			
**PNG**	0.91672*	0.9749*	0.93263*	0.90407*	0.96110*	0.93994*	0.9817*	0.95887*		

B)										

	**Brazil**	**India**	**Korea**	**Thailand**	**Turkey**					
**India**	0.68639 ns									
**Korea**	0.6184*	0.61948 ns								
**Thailand**	0.95065*	0.81079*	0.895 ns							
**Turkey**	0.0210*	0.52367 ns	0.625 ns	0.76791*						
**Singapore**	0.84141*	0.60742*	0.7 ns	0.7*	0.113 ns					

C)										

	**Brazil**	**Peru**	**Venezuela**	**China**	**India**	**Korea**	**Myanmar**	**Thailand**	**Solomon**	**PNG**
**Peru**	0.6455*									
**Venezuela**	0.6200*	0.7909*								
**China**	0.9833*	0.9925*	0.9735*							
**India**	0.8711*	0.9092*	0.8919*	0.9446*						
**Korea**	0.9851*	0.9903*	0.9744*	0.4744 ns	0.9787*					
**Myanmar**	0.8850*	0.9520*	0.9096*	0.7959 ns	0.8443*	0.8704 ns				
**Thailand**	0.9444*	0.9694*	0.9366*	0.9569*	0.8352*	0.9744*	0.7896*			
**Solomon**	0.9713*	0.9810*	0.9760*	0.9321*	0.9625*	0.8667*	0.9025*	0.9429*		
**PNG**	0.9792*	0.9846*	0.9784*	0.8444*	0.9505*	0.8914*	0.8628*	0.9368*	0.6270 ns	
**Philippines**	0.9637*	0.9958*	0.9648*	0.9746*	0.9228*	0.9913*	0.8857*	0.8689*	0.9669*	0.978*

A) DBP B) MSP-1 and C) AMA-1

## Discussion

When designing an efficient anti-malarial vaccine, worldwide information of the circulating antigenic variants is necessary for the formulation of a polyvalent vaccine, which would be effective in different regions. Because of the complex parasite life cycle and the co-existence of *P. falciparum *and *P. vivax *infections in most endemic areas, it is accepted that a multi-antigen and multi-species vaccine should be developed for most endemic regions [1]. In *P. falciparum*, the majority of haplotypes upon which current vaccines are based have been found to be present at extremely low frequencies in the global parasite population, except for *lsa-1 *and *msp-1 *haplotypes [62]. Since immune selection might be playing a role in structuring the diversity of highly polymorphic antigens, population genetic studies in endemic regions are essential for the next generation of vaccines. In this study, the information obtained from the Peruvian isolates contribute to get a better understanding of the haplotypes circulating in the Amazon basin and to compare these results with other previously reported from different parts of the world.

For the analysis of Peruvian isolates, samples form Padreococha and San Juan were considered as a single geographic population since these areas are relatively closed and they present similar haplotype distribution in all antigens with little or none genetic differentiation between them. In general, data from a two-year period (2006-2007) in Peru revealed high nucleotide and haplotype diversity compared to other geographic populations included in the final analysis. These results suggest that the extent of allelic diversity in this region is determined by complex demographic processes that include heterogeneity of transmission intensities in time and space, as well as the number of recurrent infections that are common in the region, allowing for a high turnover of parasite genotypes [41]. The presence of asymptomatic cases [64] participating in the infection process might also be contributing to the persistence of genetic variation since they are a "reservoir" of untreated patients. In addition, we need to take in consideration possible *P. vivax *multi clonal infections that might be difficult to observe by direct sequencing. This limitation could potentially affect the overall estimates of genetic diversity and population structure; however, for the goal of this study, the data collected is useful to obtain a broad picture of how these vaccine candidate haplotypes are worldwide distributed. If a fine scale study is considered in which the goal is to understand the population structure of *Plasmodium *in a specific geographic area then, unlinked microsatellite loci should be used to get a better idea of the parasite's population history.

When considering worldwide data, genes encoding non-merozoite antigens in *P. falciparum *showed variation in diversity [62]. In *P. vivax*, the same pattern was observed using CSP and TRAP. Although, limited population samples were available for TRAP sequences. On the other hand, the genes encoding merozoite antigens in *P. falciparum *and *P. vivax *showed different patterns. *P. falciparum *genes encoding AMA-1, EBA-175 and MSP-1, MSP-3 and MSP-4 showed similar levels of diversity among countries and regions [62]. In *P. vivax*, genes *dbpII *and *ama-1 *also showed this trend; however, *msp-1 *exhibited very low but different polymorphism and haplotype diversity values among regions and countries. These results support the hypothesis that maintaining the structural conformation of the HARBs regions (MSP-1_20 _and MSP-1_14_) is important for recognition and binding of reticulocytes [31]. The limited variation of amino acid substitutions in these fragments makes them strong candidates to be tested in immunological studies. Also, TRAP sequences showed low levels of nucleotide and haplotype diversity with a highly conserved region between positions 209 to 256, previously reported to be immunogenic.

Contrary to *P. falciparum*, a largely structured diversity in all *P. vivax *populations was not found on the basis of geography. The clusters determined by Structure were found in most countries except in those with small number of samples, suggesting that haplotypes corresponding to those clusters were underrepresented. Moreover, a high level of admixture in all countries was observed, which could be explained by natural and high fluctuations over time, as a result of frequency-dependent selection or the discrepancy due to the sampling used [62], since the worldwide sequences obtained from GenBank came from different time points and locations. On the other hand, the lack of geographic clustering could be explained by balancing selection acting on the genes studied and as a result high worldwide antigen diversity was observed not only in Asia but also in South America.

## Conclusion

In conclusion, it would be ideal to have a good representation of the haplotypes circulating worldwide when implementing a vaccine regardless of the geographic region of deployment. Considering that selective pressure plays an important role in structuring the diversity of antigens, it cannot be assumed that a single strain could accurately represent the haplotypes circulating worldwide and as a consequence be effective in all endemic regions. In addition, *Plasmodium *population definition should be revised since parasite sub-groups are not necessarily geographically restricted and treatment or vaccination in a region cannot be considered as if it was isolated but a dynamic map needs to be acknowledged. Thus, important epidemiological units should be established according to the parasite's gene flow, which would lead to a more effective malaria control strategy.

## Competing interests

The authors declare that they have no competing interests.

## Authors' contributions

SC conducted the molecular genetic studies, performed the genetic diversity experiments, analysed the data and wrote the first draft of the manuscript. LT performed part of the genetic diversity experiments. AE, CL, SD contributed to the write up and reviewed the final draft. DB designed the project, supervised and directed the research team and contributed to the writing and editing of the manuscript. All authors read and approved the final manuscript.
